# Interaction between ANXA1 and GATA-3 in Immunosuppression of CD4^+^ T Cells

**DOI:** 10.1155/2016/1701059

**Published:** 2016-10-19

**Authors:** Peng Huang, Yuxiang Zhou, Zan Liu, Pihong Zhang

**Affiliations:** Department of Burns and Reconstructive Surgery, Xiangya Hospital, Central South University, Changsha, Hunan, China

## Abstract

Decreased Th1/Th2 ratio is one of the major characteristics of immunosuppression in sepsis. Both membrane adhesive protein Annexin-A1 (ANXA1) and transcription factor GATA-3 have been reported to play important roles in T cell differentiation. However, the relationship between ANXA1 and GATA-3 in Th1/Th2 shift is unknown. Our study investigated the interaction effects of ANXA1 and GATA-3 to influence T cell differentiation in CD4^+^ T cells. We found that GATA-3 and ANXA1 were coexpressed on Th0/Th1/Th2 cytoplasm and nuclear. Overexpressed ANXA1 significantly increased the expression of IFN*γ* and reduced IL-4 expression in T cells, while ANXA1-silenced T cells exhibited decreased production of IFN*γ* and increased production of IL-4. Knockdown of ANXA1 promoted higher expression level of GATA-3 and low level of T-box transcription factor (T-bet/Tbx21). Further study demonstrated that ANXA1 regulated GATA-3 expression through the formyl peptide receptor like-1 (FPRL-1) downstream signaling pathways ERK and PKB/Akt. These results suggested that ANXA1 modulates GATA-3/T-bet expression induced Th0/Th1 differentiation. Moreover, we found that GATA-3 inhibited ANXA1 expression by binding to its promoter for the first time. It is proposed that the interactions between ANXA1 and GATA-3 may provide clues to understand the immunosuppression and have potential as new therapeutic targets in immunotherapy after sepsis.

## 1. Introduction

Recent clinical and experimental studies have indicated that the long-term effects of severe inflammatory events often include the suppression of immune system functions. The decrease of Th1/Th2 ratio is one of the major characteristics of immunosuppression in sepsis [1]. It is reported that membrane adhesive protein Annexin-A1 (ANXA1) and transcription factor GATA-3, which were both decreased in sepsis patients [2, 3], play important roles in the Th1/Th2 shift [4]. Many researchers have studied ANXA1 and GATA-3, respectively, but the relationship between ANXA1 and GATA-3 is still unknown. Exploring the interactions between ANXA1 and GATA-3 may provide clues to understand the immunosuppression and improve the treatment effects of sepsis patients.

As an anti-inflammatory protein, ANXA1 plays a homeostatic role in the innate immune system through mediating immune cells, such as neutrophils and macrophages [5]. Endogenous ANXA1 markedly reduced leukocyte adhesion to postcapillary venules through formyl peptide receptor (FPR) pathway [6]. Furthermore, ANXA1 promotes inflammatory cell apoptosis associated with transient rise of intracellular calcium and caspase-3 activation. Moreover, ANXA1 has been recently identified as one of the “eat me” signals on apoptotic cells to be recognized and ingested by phagocytes [7]. Studies on the expression of ANXA1 in human and mouse leukocytes have shown that this protein is also expressed at higher levels in cells of the adaptive immune response, such as T and B lymphocytes [8–10]. Further research indicates that ANXA1 increases T cells activation and favors their differentiation to Th1 cells by modulating T cell receptor (TCR) signaling [4]. In addition, analysis of the inflammatory response of ANXA1^−/−^ mice has demonstrated an exquisite role of ANXA1 in modulation of TCR signaling by the FPR family [11]. These findings suggest a potential role of ANXA1/FPRL-1 pathway in the adaptive immune response.

Upon antigen stimulation of their TCR by antigen presenting cells, naïve CD4^+^ T cells can differentiate to at least two different types of T helpers, Th1 and Th2 cells, which were documented to be involved in adaptive immunity [12]. The transcription factor GATA-3 is selectively upregulated during Th2 differentiation* in vitro* [13, 14]. GATA-3 is important not only for the transactivation of Th2 cytokine genes but also for the suppression of Th1 development [15]. GATA-3-deficient cells fail to give rise to cells of the T cell lineage [16].* In vivo* experiment from ANXA1^−/−^ mice exhibited that ANXA1-deficient T cells expressed Th2 skewing [17]. However, there is not enough evidence to support the interrelation between GATA-3 and ANXA1.

In our previous studies, the expressions of ANXA1 and GATA-3 were both decreased in the burnt mouse with sepsis [18]. The purpose of this study is to investigate whether ANXA1 and GATA-3 interact with each other to influence the immune response in T lymphocyte, as well as exploring the possible molecular mechanisms involved. Our results show that overexpressed ANXA1 (or GATA-3) represses the expression of GATA-3 (or ANXA1), while knockdown of ANXA1 (or GATA-3) increases the GATA-3 (or ANXA1) expression. Further studies indicate that ANXA1 regulates GATA-3 expression through ANXA1/FPRL-1/ERK and PKB/Akt signaling pathways, and GATA-3 mediates ANXA1 transcription activity by binding to ANXA1 promoter. Thus this study, together with our previous observations of ANXA1, suggests that the ANXA1/FPRL-1 axis and GATA-3 are potential therapeutic targets of the Th1/Th2-mediated immunological suppression in sepsis.

## 2. Materials and Methods

### 2.1. Reagents

Anti-mouse CD3e (clone 145-2c11) and anti-mouse CD28 (clone 37.51) were purchased from BD Bioscience (San Jose, CA). Murine IL-2, IL-4, IFN*γ*, IL-12, anti-IL-4 (clone 11B11), and anti-IFN*γ* (clone XMG1.2) were purchased from eBioscience (Wembley, United Kingdom). Anti-mouse GATA-3, total and phosphorylated ERK1/2, and AKT were purchased from Cell Signaling (Danvers, MA). Anti-T-box transcription factor (T-bet/Tbx21) was purchased from Abcam (Cambridge, UK), anti-FPRL-1 purchased from NOVUS (Colorado, USA), and anti-ANXA1 purchased from Proteintech (Manchester, UK). Unless otherwise specified, all the other reagents were from Sigma-Aldrich (St. Louis, MO).

### 2.2. Mice

KM male mice with weight of 35–50 g were obtained from Department of Experimental Animals, Central South University (Changsha, China). Animal work has been performed according to the approval of the ethics of Xiangya Hospital, Central South University.

### 2.3. Cell Culture

Mice were anesthetized with sodium pentobarbital [40–50 mg/kg intraperitoneal (i.p.)]. Their spleen was teased apart to make a single-cell suspension and stained with APC-conjugated anti-CD4. Then the CD4^+^ cells were purified by FACS (BD Aria III). The isolated naive CD4^+^ T cells (>95%, 1 × 10^6^/6 wells) were cultured in RPMI 1640 (GIBCO, Grand Island, NY) complete medium. The culture conditions of T cells are as follows: Th0: anti-CD3/CD28 (5 *μ*g/mL) and IL-2 (50 U/mL); Th1: IL-12 (2 ng/mL), IL-2 (50 U/mL), and anti- IL-4 (20 *μ*g/mL) and Th2: IL-4 (1000 U/mL), IL-2 (50 U/mL), and anti-IFN*γ* (10 *μ*g/mL).

### 2.4. Vector Constructs and* Lentivirus* Production

The sequences for ANXA1 and GATA-3 overexpression were artificially synthesized and cloned into the PLV (Exp) Puro-IRES-EGFP vector (Cyagen, Guangzhou, China).

Interference lentiviral (pLent iX1 Puro-eGFP vector) against ANXA1 and GATA-3 was constructed. Three candidate shRNA sequences targeting ANXA1 and GATA-3 were designed and synthesized by Cyagen. All virus packing was performed in 293T cells after the cotransfection.* Lentivirus* construction was performed using the Lipofection 2000 reagent (Invitrogen). Viruses were harvested 48 h after transfection, and viral titers were determined.

### 2.5. Cell Transfection

The above constructed* Lentivirus* was stably transfected into the T cells with an optimal multiplicity of infection (MOI) of 100 TU/mL after stimulated with anti-CD3/CD28 (5 *μ*g/mL) for 24 h. Upregulated (downregulated) expressions of ANXA1 and GATA-3 were confirmed by qRT-PCR and western blot (see Supplementary Figure 1 in Supplementary Material available online at http://dx.doi.org/10.1155/2016/1701059). The shRNA sequences with the maximum of more than 75% knockdown efficiency were selected for further stable ANXA1 and GATA-3 silencing in T cells. The most efficient ANXA1 shRNA sequence of three was constructed as follows: (shANXA1-3) 5′-GAGATCTGGCCAAAGACATAA-3′; (anti-shANXA1-3) 5′-TTATGTCTTTGGCCAGATCT-3′. The most efficient GATA-3 shRNA sequence of three was constructed as follows: (shGATA-3-2) 5′-GCTCAGTATCCGCTGACGGAA-3′; (anti-shGATA-3-2) 5′-TTCCGTCAGCGGATACTGAGC-3′.

### 2.6. Cytokine ELISA

Th0 cells (1 × 10^6^/mL), obtained after infection with UPANXA1 and culturing in Th0 conditions with complete RPMI medium for seven days, were stimulated with phorbol 12-myristate 13-acetate(PMA) 10 ng/mL and ionomycin 1 *μ*g/mL for 4 h in 6-well plates to produce cytokine [18]. Culture supernatants were collected and analyzed for IFN*γ* and IL-4. They were measured to reflect Th1/Th2 reactions. The IFN*γ* and IL-4 levels were measured by ELISA kits purchased from Multi-Sciences (China). The assay was carried out according to the manufacturer's protocol.

### 2.7. RNA Extraction and Real-Time PCR

qRT-PCR was performed to determine the expression levels of GATA-3, T-bet, and ANXA1. Total RNA extraction was performed using simply P total RNA extraction kit (Bioflux, Europe). The cDNA synthesis was performed using All-in-One™ First-Strand cDNA Synthesis Kit (GeneCopoeia, Maryland, USA). cDNA was used to set up a quantitative real-time PCR (qPCR) reaction using the All-in-One qPCR Mix (GeneCopoeia). The expression levels of GATA-3, ANXA1, and T-bet were normalized to actin. The primer sequences were as follows: mouse GATA-3 forward primer: 5′-GCTGGATGGCGGCAAAG-3′, mouse GATA-3 reverse primer: 5′-GTGGGCGGGAAGGTGAA-3′, mouse ANXA1 forward primer: 5′-AAGGTGGTCCTGGGTCAGC-3′, mouse ANXA1 reverse primer: 5′-TGAGCATTGGTCCTCTTGGT-3′, mouse Tbx21/T-bet forward primer: 5′-ATGTTCCCATTCCTGTCCTTCA-3′, mouse Tbx21/T-bet reverse primer: 5′-AAATGAAACTTCCTGGCGCATC-3′, mouse actin forward primer: 5′-CATCCTGCGTCTGGACCTGG-3′, and mouse actin reverse primer: 5′-TAATGTCACGCACGATTTCC-3′. All protocols were carried out according to the manufacturer's instructions. Each sample was run in triplicate.

### 2.8. Western Blotting

Total protein was extracted using lysis buffer. 30 *μ*g protein was separated by SDS-PAGE, transferred onto PVDF membrane, and hybridized with a primary antibody followed by a horseradish-conjugated secondary antibody. Detection of *β*-actin on the same membrane was used as a loading control.

### 2.9. Immunofluorescence Staining

Th0/Th1/Th2 cells were washed with ice-cold PBS and fixed with 4% paraformaldehyde. The cells permeabilized with 0.1% Triton X-100 were incubated with 2% bovine serum albumin for 30 min and followed by the primary antibodies against GATA-3 and ANXA1 at 4°C overnight. The slides of cells were subsequently incubated with the corresponding Alexa Fluor 488-conjugated secondary antibodies for 1 h at room temperature. Nuclei were stained with DAPI for 3 min. Samples were examined to analyze the expression of GATA-3 and ANXA1.

### 2.10. Promoter Dual Luciferase Reporter Assay

The GATA-3 pENTER (NM-001002295) and control pENTER plasmids were obtained from Vigene Biosciences (Rockville, MD, USA). The PGL3 enhancer vector and PRL-TK vector were obtained from Promega (Madison, WI, USA). ANXA1 (NM00200.2) promoter region 2000 bp base-pairs and firefly luciferase reporter gene were built in PGL-3 enhancer vector and the renilla luciferase reporter gene was built in PRL-TK vector. The renilla luciferase plasmid was used in all the experiment to normalize the efficiency of the transfection. Transient transfection of 293T cells was performed as previously described. Briefly, on the day of transfection, 5 × 10^4^ cells were plated in 100 *μ*L in each well of a 96-well pate prior to incubation with VigeneFection (Vigene, Rockville, MD) complexes according to the manufacturer's instruction using a total of 0.3 *μ*g DNA and 0.9 *μ*L VigeneFection. At 48 to 72 hours after transfection, cells were lysed in 20 *μ*L reporter lysis buffer (Promega, Madison, WI) for 15 minutes on ice. Add the 100 *μ*L Luciferase Assay Reagent II (Promega) and record the firefly luciferase activity measurement. And then add the 100 *μ*L STOP & GLO^®^ Reagent (Promega) and read the renilla luciferase activity measurement. At least three independent experiments were performed and the data were presented as mean ± SEM.

### 2.11. Statistical Analysis

All data were presented as mean ± SEM and analyzed by Student's *t*-test. *p* < 0.05 was considered to be statistically significant.

## 3. Result

### 3.1. ANXA1 and GATA-3 Expression on CD4^+^ T Cells

**Figure 1:**
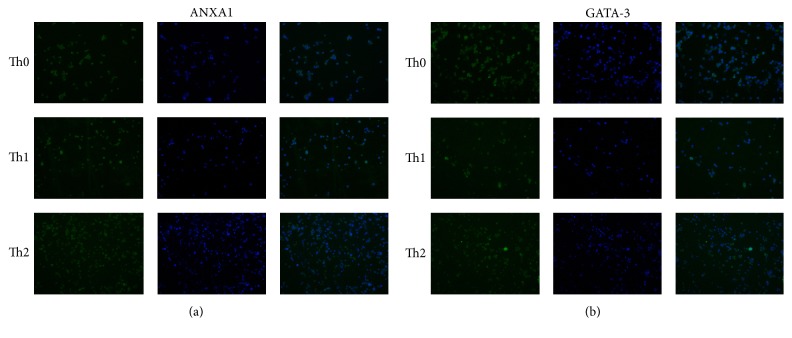
ANXA1 and GATA-3 expression on Th0, Th1, and Th2 and localization on cytoplasm and membrane. (a) Immunofluorescence analysis of ANXA1 expression on Th0, Th1, and Th2. Immunofluorescence was carried out using green fluorescent tagged secondary anti-ANXA1. The cell nuclei were stained with DAPI. (b) Immunofluorescence analysis of GATA-3 expression on Th0, Th1, and Th2. Immunofluorescence was carried out using green fluorescent tagged secondary anti-GATA-3. The cell nuclei were stained with DAPI.

To explore the expression of ANXA1 and/or GATA-3 of the Th0, Th1, and Th2 cells, immunofluorescence was carried out to confirm that ANXA1 and GATA-3 expression were located in cell nucleus and cytoplasm (Figure 1).

### 3.2. ANXA1 Modulates GATA-3/T-bet Expression

**Figure 2:**
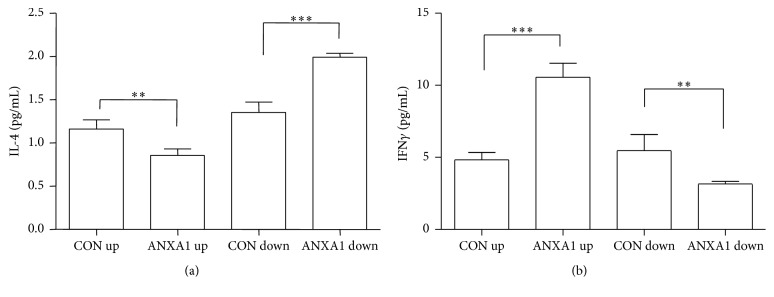
ANXA1 modulates the differentiation of CD4^+^ T cells in Th0 conditions. CD4^+^ T cells were infected with ANXA1 up or ANXA1 down* Lentivirus* in Th0 condition and restimulated with PMA 10 ng/mL and ionomycin 1 *μ*g/mL for 4 h. Then Th1 (IFN*γ*) or Th2 cytokine (IL-4) production was to be measured. Values are mean ± SEM of *n* = 3. ^*∗∗*^ *p* < 0.01. ^*∗∗∗*^ *p* < 0.001.

To determine the roles of overexpressed endogenous ANXA1 in the balance between GATA-3 and T-bet expression, two major transcriptional switches in Th1/Th2 differentiation, we examined the differentiation of T cells, which is driven by the strength of overexpressed ANXA1 in Th0 conditions. Naive T cells from mouse were infected with UPANXA1 viruses and cultured in Th0 medium for seven days, followed by being exposed to PMA (10 ng/mL) and ionomycin (1 *μ*g/mL) for 4 h to stimulate the production of cytokines in Th1/Th2 [1]. UPANXA1 significantly increased the expression of IFN*γ* and reduced IL-4 expression in T cells, compared with those infected with up-control (*p* < 0.01, Figure 2). ANXA1-silenced T cells exhibited decreased production of IFN*γ* and increased approximately 50% higher production of IL-4, compared with T cells infected with down-control (*p* < 0.01, Figure 2). These results demonstrated that ANXA1 induced Th0/Th1 differentiation, whereas cells infected with silenced ANXA1 promoted Th0/Th2 differentiation.

**Figure 3:**
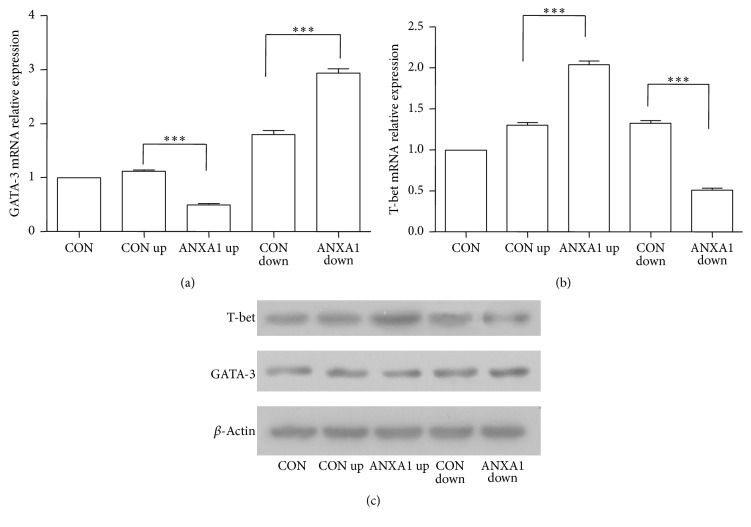
ANXA1 modulates GATA-3 and T-bet expression in Th0 conditions. CD4^+^ T cells were infected with ANXA1 up or ANXA1 down* Lentivirus* in Th0 condition and then stimulated with anti-CD3/CD28 (5.0 *μ*g/mL) for 8 h to analyze T-bet and GATA-3 expression by real-time PCR (a, b) and western bolt (c). ^*∗∗∗*^ *p* < 0.001.

The important roles of GATA-3/T-bet in influencing differentiation of T cell lineage into Th1 or Th2 effector cells were well accepted [19, 20]. We hypothesized that ANXA1 could modulate GATA-3/T-bet expression to affect Th1/Th2 differentiation. As shown in Figure 3, the results indicated that ANXA1-silenced T cells expressed higher levels of GATA-3 and low level of T-bet, compared with the control and UPANXA1 groups (*p* < 0.01).

### 3.3. ANXA1 Modulates GATA-3 Expression through FPRL-1 Signaling Pathways

**Figure 4:**
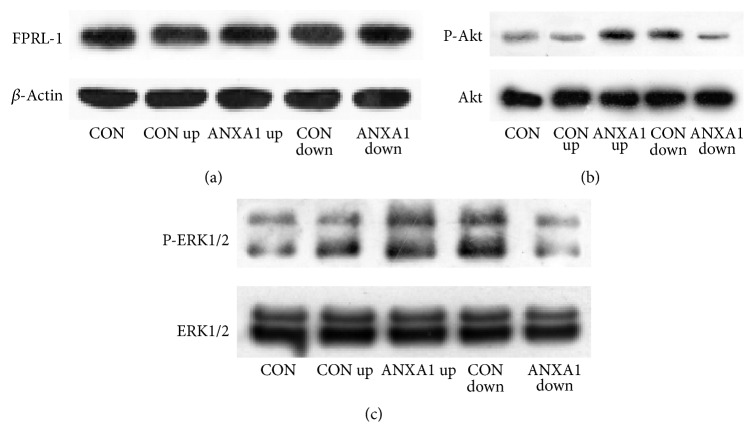
ANXA1 modulates GATA-3 expression through ERK and AKT pathway. CD4^+^ T cells were infected with ANXA1 up or ANXA1 down* Lentivirus* in Th0 condition and then stimulated with anti-CD3/CD28 (5.0 *μ*g/mL) for 8 h to analyze FPRL-1, ERK, and Akt expression by western blot.

FPRL-1 is one receptor of ANXA1. Considering that ERK and PKB/Akt are two major downstream transcription factors of FPRL-1, we further investigated the role of FPRL-1 signaling pathways in the regulation of ANXA1 on GATA-3. The result showed that ANXA1 significantly phosphorylated ERK and PKB/Akt, while knockdown of ANXA1 decreased ERK and Akt activation. The expression levels of FRRL-1 in all groups, regardless of ANXA1 status, were not significantly different (Figure 4).

### 3.4. GATA-3 Regulates ANXA1 Expression in CD4^+^ T Cells

**Figure 5:**
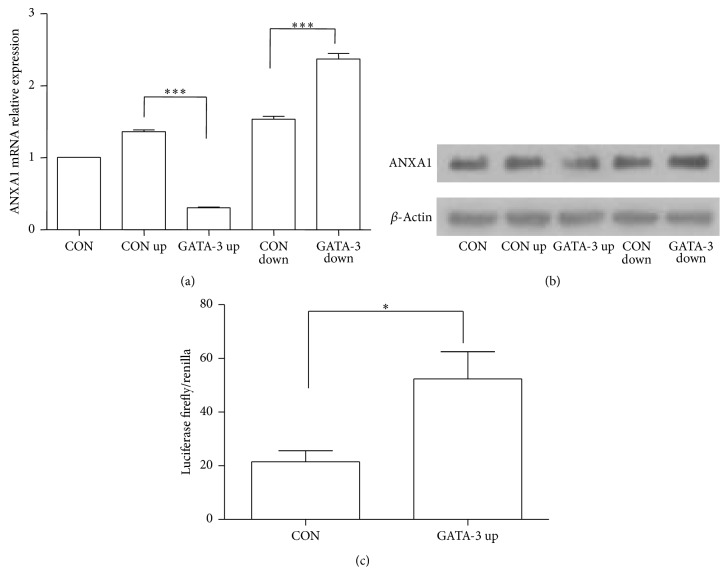
GATA-3 modulates ANXA1 expression in Th0 conditions. CD4^+^ T cells were infected with GATA-3 up or GATA-3 down* Lentivirus* in Th0 condition and then stimulated with anti-CD3/CD28 (5.0 *μ*g/mL) for 8 h to analyze ANXA1 expression by real-time PCR (a) and western bolt (b). Dual luciferase assays showed an increase after transfection of GATA-3 in 293T cells (c). ^*∗*^ *p* < 0.05. ^*∗∗∗*^ *p* < 0.001.

To investigate the effect of GATA-3 on ANXA1 expression in CD4^+^ T cells, CD4^+^ cells with different GATA-3 expression levels were used. As shown in Figures 5(a) and 5(b), overexpressed GATA-3 inhibited ANXA1 protein and mRNA expression, while silenced GATA-3 increased the expression of ANXA1.

### 3.5. GATA-3 Regulates ANXA1 Expression through Binding to ANXA1 Promoter in 293T Cells

As a transcription factor, GATA-3 mediates ANXA1 expression through ANXA1 promoter. ANXA1 promoter area about 2000 bp was found in the Eukaryotic Promoter Database and UCSC Genome Bioinformatics. The JASPAR database was used to predict the binding domain of ANXA1 promoter with GATA-3. The result suggested that GATA-3 could combine ANXA1 promoter AGATAAGA (start-281 end-274) area on the level of nucleic acid sequences. The area the same as zinc finger of GATA-3 closet to the C terminus binds to the consensus DNA sequence area (A/T)GATA(A/G) [21]. To verify this hypothesis, ANXA1 promoter area (2000 bp) and luciferase reporter gene were built in PGL-3 enhancer vector and renilla luciferase reporter gene was built in PRL-TK. Three plasmids GATA-3 pENTER, ANXA1 promoter PGL3, and PRL-TK were transfected in 293T cell. As shown in Figure 5(c), the luciferase activity was significantly increased, compared with up-controls when cotransfected with UPGATA-3. These results demonstrate that GATA-3 binds to ANXA1 promoter to repress its expression in 293T cells.

## 4. Discussion

Sepsis is defined as a systemic inflammatory response to infection. Excessive inflammatory responses including the release of a cytokine storm and the activation of a large number of T cells are characteristics of early stage sepsis [22]. In spite of their ability to kill pathogens, they are equally effective in inducing cell and tissue damage [23]. On one hand, the cytokine storm could be protective by activating the host anti-inflammatory proresolving response [24]. On the other hand, during sepsis, the cytokine storm induces excessive activation induced T cell death (AICD); the cause of AICD is an aberration in the cell cycle program [25]. Enhanced anti-inflammatory response, characterized by a large number of T cells apoptosis and decreased responsiveness to antigen/mitogen, leads to the immunosuppression.

Immunosuppression after severe sepsis remains the main cause of mortality [26]. More patients die in the immunosuppression phase where the risk of a secondary infection increases. Anti-inflammatory therapy can improve the survival rate of patients in hyperinflammatory status, while it may be worse if applied during the sepsis-induced immunosuppression [27]. The conventional understanding of immune responses of post-sepsis involves a shift away from Th1 responses towards Th2 responses [1]. A low ratio of Th1/Th2 is a feature of immunosuppression [28]. Promoting a low ratio of Th1/Th2 immune responses to Th1 skewing might be a treatment of immunosuppression.

Studies have shown that ANXA1 is an endogenous anti-inflammatory factor in the innate immune system [6, 29]. Increasing evidences suggest that ANXA1 plays an important role in the regulation of the adaptive immune response. ANXA1 could modulate Th1/Th2 differentiation by activating TCR signaling [4, 30]. Consistent with above studies, we found that overexpressed ANXA1 induced the differentiation of Th1/Th2 to Th1 skewing with increased IFN*γ* and reduced IL-4 cytokines expression. In the process of Th1/Th2 differentiation, another important factor is GATA-3. As a specific transcription factor of Th2 [14], GATA-3 activates TCR signaling in pre-T cells and promotes the CD4^+^ T cell lineage differentiation after positive selection [31].

Many researches and our previous studies have found that the ANXA1 plasma levels were decreased in the rabbit and patients with severe sepsis [2, 32]. Another study demonstrated low expression level of GATA-3 in septic shock [3]. However, the reports about the correlations between ANXA1 and GATA-3 are limited. Our previous animal model observed that ANXA1 and GATA-3 both present the same downtrend in mouse with severe sepsis [18, 33]; this phenomenon may be related with a large number of T cells apoptosis. But the specific regulatory mechanism between them is not clear. The major finding of this study is the discovery of a novel role of transcription factor GATA-3 regulation on ANXA1 in the adaptive immune response and the molecular mechanisms of mutual regulation contributing to immunosuppression in inflammatory processes.

Our results indicated that expressions of ANXA1 and GATA-3 are both located in Th0, Th1, and Th2 cells nucleus and cytoplasm. Oliani et al. also found that ANXA1 was expressed in the eosinophil cytosol and nucleus [34]. Further study manifested that overexpressed endogenous ANXA1 exerted an effect on decline of GATA-3 expression and increase of T-bet expression, which may be one reason of ANXA1 inducing the differentiation of Th1/Th2 to Th1 skewing. One viewpoint deems that ANXA1 modulates Th1/Th2 differentiation by promoting T cell activation [4, 30]. But another study finds T cell-expressed ANXA1 inhibits T cell activation in the adaptive immune response [35]. Our view is that T cells differentiate into various subtypes according to environmental signals [36], and ANXA1 modulates T cell differentiation through modulating their transcription factors.

It is known that ANXA1 is one endogenous ligand of FPRL-1, which belongs to the members of the G protein-coupled receptor family. Exogenous ANXA1 protein and its N-terminal peptide Ac2-26 were demonstrated to activate ERK* in vitro* and* in vitro* [37].

It is reported that ERK suppresses many T cell differentiation related transcription factors, including GATA-3 and FOXP3 in the T cells [33, 38]. Interestingly, some studies show that ERK-MAPK cascade controls GATA-3 stability through the ubiquitin/proteasome-dependent pathway. These studies further verify the important roles of activated ERK-MAPK cascade in the GATA-3 phosphorylation and its ubiquitin-mediated degradation through the 26 S proteasome [39]. So ERK-MAPK is essential for GATA-3 activation and stability to avoid GATA-3 excessive expression in stimulated T cells. Meanwhile, ERK pathway is also essential for T cell proliferation and Th1 skewing by inducing the Tbx21 expression [33]. Akt is a GATA-3 phosphorylase kinase and the activation of Akt1 induces derepression of Tbx21 and IFN*γ* expression in Th2 cells. Akt phosphorylates GATA-3 and induces the dissociation of Hdac2 from the GATA-3 complex, resulting in a failure in the repression of IFN*γ* expression in Th2 cell. These findings highlight a pivotal role of posttranscriptional modification of GATA-3, which is a kind of negative feedback regulation mechanism of Th2 differentiation to avoid excessive differentiation to Th2 cell [40]. Therefore, it is proposed that the enhanced ANXA1 regulates GATA-3 expression to induce Th1/Th2 shift by ERK and PKB/Akt to influence the inflammatory response and balance the early proinflammatory function.

Another interesting discovery in this study is that the GATA-3 also downregulated ANXA1 expression in CD4^+^ T cells. Further research indicated that GATA-3 exerted a transcriptional-level control by combining with the promoter of ANXA1 in 293T cells. This experimental conclusion needs to be verified in CD4^+^ T cells and determined further by Electrophoretic Mobility Shift Assay or Chromatin Immunoprecipitation.

In the early sepsis stage, TCR trigger GATA-3 upregulation* via* PI3K-mTOR dependent pathways [41]. Moreover, GATA-3 is suggested to mediate notch signaling that is important for promoting T cell development and proliferation [42]. In our studies, if we downregulate GATA-3 expression, the level of ANXA1 expression increased. On one hand, it can remit Th1/Th2 toward to Th2 skewing to prevent immunosuppression. On the other hand, as an anti-inflammatory protein, ANXA1 can balance the early proinflammatory function.

In the immunosuppression stage, GATA-3 is a potential marker of recovery [3]. If we upregulate the GATA-3 expression, the expression level of ANXA1 decreases. GATA-3 can strengthen the TCR activation to improve adaptive immune [43] and decreased ANXA1 can reduce anti-inflammatory response at a certain extent for better care of patients.

In conclusion, our results suggest that GATA-3 binds to ANXA1 promoter area to inhibit ANXA1 expression and functions. ANXA1-mediated GATA-3 and T-bet might favor Th1 differentiation through ERK and AKT pathway, reversing tendency characterized by Th2 skewing of immunosuppression. Based on the Th1/Th2 mediation, our discoveries might provide a potential treatment of immunosuppression after sepsis.

## Supplementary Material

UP-regulated and Down-regulated expressions of GATA-3(ANXA1) were constructed by lentiviral and confirmed by Western blot (A,B) and qRT-PCR (C,D). The most efficient GATA-3 shRNA sequence of three was GATA-3 DOWN 2 and the most efficient ANXA1 shRNA sequence of three was ANXA1 DOWN3.

## Abbreviations

ANXA1: Annexin-A1
T-bet (Tbx21): T-box transcription factor
TCR: T cells receptor
FPR: Formyl peptide receptor
FPRL-1: Formyl peptide receptor like-1
PMA: Phorbol 12-myristate 13-acetate
Th0/1/2: T helper type 0/1/2.
