# Supplementary figures and images for: Interaction between ANXA1 and GATA-3 in Immunosuppression of CD4+ T Cells
Source: Mediators Inflamm. 2016 Oct 19;2016:1701059. doi: 10.1155/2016/1701059 (PMC5090097; doi:10.1155/2016/1701059)

Supplementary Figure 1

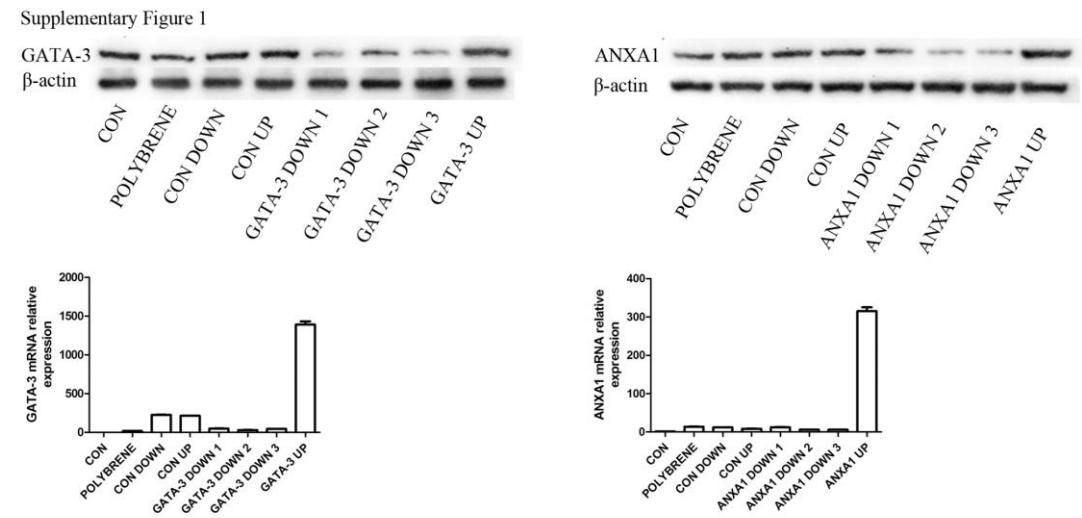

Supplement: Supplementary file 1 — UP-regulated and Down-regulated expressions of GATA-3(ANXA1) were constructed by lentiviral and confirmed by Western blot (A,B) and qRT-PCR (C,D). The most efficient GATA-3 shRNA sequence of three was GATA-3 DOWN 2 and the most efficient ANXA1 shRNA sequence of three was ANXA1 DOWN3. [file 1701059.f1.pdf]
